# Seroprevalence of *Toxoplasma gondii* Infection in Wild Boar (*Sus scrofa*) and Korean Water Deer (*Hydropotes inermis argyropus*) in the Republic of Korea

**DOI:** 10.3390/ani14243669

**Published:** 2024-12-19

**Authors:** Jusun Hwang, Jisoo Kim, Kidong Son, Yongkwan Kim, Hyesung Jeong, Weonhwa Jheong

**Affiliations:** National Wildlife Institute of Wildlife Disease Control and Prevention, 1, Songam-gil, Gwansan-gu, Gwangju 62407, Republic of Korea; jusunhwang@aware.kr (J.H.); jskim89@korea.kr (J.K.); skd9326@korea.kr (K.S.); kyk5388@korea.kr (Y.K.); halley@korea.kr (H.J.)

**Keywords:** *Toxoplasma gondii*, seroprevalence, wild boar, Korean water deer

## Abstract

*Toxoplasma gondii* is a ubiquitous zoonotic parasite with a wide range of warm-blooded animals as intermediate hosts, where the transmission primarily occurs through ingesting *T. gondii* oocysts in the environment. Hence, animals inhabiting a wide geographical range can be sentinels for the environmental contamination of *T. gondii* oocysts. The aim of this study was to determine the seroprevalence of *T. gondii* in two generalist ungulates in the Republic of Korea (ROK), wild boar (*Sus scrofa*) and Korean water deer (*Hydropotes inermis argyropus*), and identify the risk factors associated with this infection. Commercial ELISA kits confirmed a seroprevalence of 34.9% in wild boar (170/487, 95% CI: 30.7–39.3%) and 29.9% in Korean water deer (135/452, 95% CI: 25.7–34.3%). In both species, the seroprevalence was highest in winter, and showed no sex-related differences. In wild boars, the seroprevalence showed steep reduction in 2020 and increased with body mass. Meanwhile, in Korean water deer, seroprevalence did not show significant association with any of the tested variables, although an increase in seroprevalence was observed in 2020 compared to previous years. This study documents the widespread yet heterogeneous nature of *T. gondii* prevalence in its intermediate host population. Hence, the constant monitoring of *T. gondii* prevalence in its host species, both definitive and intermediate, while simultaneously considering risk factors would be necessary to identify the drivers behind the fluctuating pattern of *T. gondii* prevalence.

## Simple Summary

*Toxoplasma gondii* is a protozoan zoonotic parasite that is endemic throughout most parts of the world. *T. gondii* utilizes a wide range of warm-blooded animals as intermediate hosts, which are commonly infected by ingesting *T. gondii* oocysts on environmental contaminants. Hence, widely distributed wild animals, such as deer and wild boar, may function as an indicator for contaminated environments. In this study, we estimated the seroprevalence of *T. gondii* and identified the related risk factors in wild boar (*Sus scrofa*) and Korean water deer (*Hydropotes inermis argyropus*), which are the two most abundant wild ungulates in the Republic of Korea (ROK). Overall seroprevalence was 34.9% in wild boar and 29.9% in Korean water deer. In both species, the seroprevalence was influenced by season, but not by sex. In wild boars, *T. gondii* seroprevalence was also strongly associated with the year and body mass. The results from this study suggest that *T. gondii* is endemic in both of the studied species and that the prevalence may vary in association with relevant factors such as sex, season, and year. Hence, constant monitoring will be required to gain information on the distribution of and fluctuation in *T. gondii* prevalence in wild hosts, which will be useful when educating the hunting community regarding the danger of parasite exposure when dealing with wild boar carcasses.

## 1. Introduction

*Toxoplasma gondii* is a protozoan parasite found worldwide and is a well-known etiological agent of toxoplasmosis in both human and animals [1]. According to FAO-WHO, *T. gondii* is ranked as the fourth most significant food-borne parasite threatening public health [2]. Toxoplasmosis can be especially problematic for those with compromised immunity and/or in the early stages of pregnancy [3]. Meanwhile, *T. gondii* can also have a detrimental economic impact by causing abortions in livestock, i.e., pigs, cattle, and equids [4]. *T. gondii* has a complex life cycle, with a wide range of warm-blooded animals acting as intermediate hosts, whereas only feline species can serve as definitive hosts. After sexual reproduction within the definitive host, parasite oocysts are shed through feces and undergo sporulation [1]. Sporulated oocysts are highly resistant in the external environment and are known to survive in the soil for over 18 months within a wide temperature range, although their survival can be lowered in dry conditions and/or extreme temperatures [5,6]. As such, *T. gondii* oocysts persist in the environment, functioning as the main infection source for intermediate hosts through ingestion. In wildlife hosts, the seroprevalence of *T. gondii* has been frequently investigated in generalist ungulates, such as wild boar and roe deer [7,8,9], since these species are capable of using a wide range of habitat types and may serve as sentinels for environmental *T. gondii* oocyst contamination [10,11].

In the Republic of Korea (ROK), wild ungulates that can be considered as generalist species include Korean water deer (*Hydropotes inermis argyropus*) (hereafter KWD) and wild boar (*Sus scrofa*). Owing to its global distribution, the prevalence of *T. gondii* in wild boar has been frequently studied in many countries [12,13], including the ROK. On the other hand, empirical data regarding *T. gondii* prevalence in KWD are scarce. However, these two species differ in various ecological aspects, including diet, social structure, and space use, which can in turn affect the interaction of each species, as a potential host, with *T. gondii* oocysts in the environment [14,15,16]. Hence, information regarding the infection status of *T. gondii* in its population would allow for a better understanding of the roles and/or efficiency of each species as intermediate hosts of *T. gondii.*

Despite being listed as “vulnerable” in IUCN conservation status, KWD is considered as a common species in Korea due to its lack of natural predators, and both KWD and wild boar are designated as pest species due to their seasonal use of agricultural crops and are thus regularly hunted for population control. The meat and internal organs of both animals are often considered delicacies, especially in the rural parts of the country, although the consumption of such products is considered risky behavior [17,18]. Here, our objective was to investigate and compare the seroprevalence of *T. gondii* in the two most common ungulates, wild boar and KWD, in the ROK.

## 2. Materials and Methods

Blood samples were collected for 4 years from 2017 to 2020 in 9 provinces through opportunistic sampling from legally hunted animals (wild boar, n = 487) or wildlife rescue centers (KWD, n = 452). Hunting was carried out only by hunters with a license allowing them to hunt during the regular hunting season or as part of crop damage management. At each capture, sex and body mass were recorded and whole-blood samples were collected from the thoracic cavity or axillary veins of the hunted animals and placed in EDTA tubes and serum separation tubes. Samples from rescue centers were collected during diagnosis procedures by veterinarians. Blood samples in serum tubes were centrifuged at 2000 rpm for 15 min for serum separation and stored at −70 °C prior to analysis. Information on geographic locations, date of sampling, sex, and body mass (only in wild boars) was also recorded for each sample. Wild boars were grouped by body mass into four categories for further analysis (Table 1).

Each serum sample was tested using a commercial ELISA kit, ID Screen^®^ *Toxoplasma gondii* Indirect Multispecies ELISA (IDVet^®^, Montpellier, France), to detect *Toxoplasma gondii* antibodies. The tests were performed according to the manufacturers’ instructions.

The results were read with an ELISA plate reader (Infinite^®^200 PRO, Tecan, Männedorf, Switzerland) at 450 nm. Briefly, samples were diluted (1:10) for analysis, and the average of the optical densities (OD) of the positive controls and the difference between the averaged ODs of positive and negative control sera were calculated to provide an interpretation. As recommended by the manufacturers, serum samples with S/P percentages > 50% were determined as positive.

Statistical analyses were performed using R 3.5.0 software (R Development Core Team, 2012, Vienna, Austria). We tested for significant differences in *T. gondii* seroprevalence based on species, sex, province, and season with each species and body mass (only in wild boars) through chi-square tests with a Bonferroni correction for each single comparison. The value of *p* ≤ 0.05 was considered statistically significant. Odds ratios (ORs) were determined to assess the strength of the correlations.

## 3. Results

Based on the serological examination, we observed *T. gondii*-specific antibodies in 34.9% (170/487, 95% CI: 30.7–39.3%) of wild boars and in 29.9% (135/452, 95% CI: 25.7–34.3%) of KWD. The difference in seroprevalence between the two species (*p* = 0.11) and between the sexes was insignificant in both species (wild boar; *p* = 0.23, KWD; *p* = 0.43). Meanwhile, seroprevalence among the sampled years was significantly different in wild boars (wild boar; *p* < 0.00, KWD; *p* = 0.27), with seroprevalence dropping to 20.6% in 2020, compared to 41.7% and 41.4% in 2017 and 2019, respectively.

Meanwhile, seroprevalence in KWD ranged between 26 and 30% during the 2017–2019 period but showed an increase in 2020 to 39.7%. In wild boars, seroprevalence also showed significant variation (*p* < 0.00) among seasons, with the highest antibody prevalence in winter (44.4%) and the lowest in summer (24.6%). KWD also showed the highest seroprevalence in winter and spring, but overall did not show any significant difference across the seasons (*p* = 0.82). In wild boars, the seroprevalence increased in correlation with the body mass (*p* < 0.00), with individuals over 300 kg showing the highest prevalence (47.9%).

**Figure 1:**
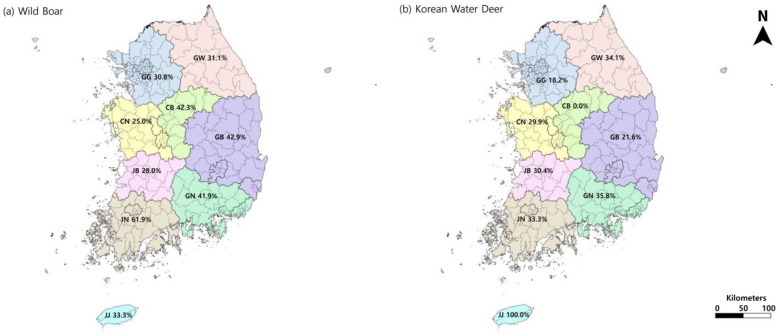
Seroprevalence of *T. gondii* by province: (**a**) wild boar; (**b**) Korean water deer (KWD).

Geographically, *T. gondii* was detected in all nine provinces investigated. Among the provinces, for wild boar, the highest seroprevalence was found in Jeonnam (13/21; 61.9%), followed by Gyeongbuk (24/56; 42.9%), Chungbuk (11/26; 42.3%), Gyeongnam (18/43; 41.9%), Gangwon (51/164; 31.1%), Gyeongii (41/133; 30.8%), Jeonbuk (7/25; 28.0%), Chungnam (4/16; 25.0%) (Table 2, Figure 1). In KWD, the highest rate was found in Gyeongnam (34/95; 35.8%), Gangwon (30/88; 34.1%), Jeonnam (14/42; 33.3%), Jeonbuk (17/56; 30.4%), and Chungnam (23/77; 29.9%), followed by Gyeongbuk (8/37; 21.6%), Gyeongi (8/44; 18.2), Chungbuk (0/12, 0.0%) (Figure 1). Only three wild boar samples and one KWD sample were collected from Jeju Island (JJ). Among these four samples, one of the three wild boar samples and the single KWD sample were positive for *T. gondii* antibody testing. In both species, the difference in seroprevalence among regions was not statistically significant (wild boar; *p* = 0.16, KWD; *p* = 0.25). Overall seroprevalence among regions ranged between 25.0 and 61.9% in wild boar and between 18.2 and 35.8% in KWD. Nevertheless, for KWD, regions with a seroprevalence of 0% (CB, n = 12) and 100% (JJ, n = 3) were also identified (Figure 1).

## 4. Discussion

This study was part of a nationwide, ongoing surveillance investigating a set of wildlife-related pathogens, including *T. gondii,* in Korean endemic wild species. As the two most common wild ungulates in the ROK, wild boar and KWD can serve as sentinel host species for the environmental contamination of *T. gondii* oocysts in wild habitats, in addition to acting as potential sources of human infection with *T. gondii* through consumption.

Our research indicated the endemic nature of *T. gondii* in both species, with a seroprevalence of 34.9% in wild boar and 29.9% in KWD. The higher seroprevalence in wild boars compared to KWD agrees with results from previous studies [7,19,20], where wild boars, as an omnivorous species, tend to show a higher prevalence than sympatric cervid species, potentially due to their rooting behavior and diet composition, which is largely composed of on-ground fruits and seeds [9,21]. The occasional scavenging of carcasses may also contribute to higher *T. gondii* seroprevalence in wild boar than cervid hosts [22,23].

In the case of wild boar, our result (34.9%) corresponds to previous reports that the prevalence of analyzed antibodies against *T. gondii* using ELISA in wild boar ranged between 19.8 and 56.7% [14]. Nevertheless, when the results are broken down by year, prevalence during 2017 and 2019 (41.4% in 2017, 41.7% in 2019) fall closer to the higher range, while the seroprevalence in 2020 (20.6%) is at the lower end of the overall range, showing the large yearly variation in seroprevalence in this study. Two studies from the ROK that analyzed samples of wild boar collected between 2009 and 2011 and between 2008 and 2012 yielded seroprevalence of 25.1% and 36.0%, respectively. However, the annual prevalence was not reported, rendering the comparison of the temporal pattern with other studies difficult [17,24]. To our best of knowledge, the yearly variation in *T. gondii* seroprevalence in wild boars has rarely been explored. This may partly be because many surveillance efforts were based on samples collected in a single year or a single hunting season, which limits the chances of identifying the existence of temporal fluctuations in *T. gondii* prevalence in its host population and the drivers behind this pattern [7,25,26]. In this study, a sudden drop in seroprevalence was observed in wild boars from 41.4% in 2019 to 20.6% in 2020. In wild boars, the transmission of *T. gondii* is known to be density-dependent, potentially in relation to the extent of environmental oocyst contamination and the scavenging of conspecifics [14,19,27]. In the ROK, the wild boar population size is suspected to be reducing dramatically due to an African swine fever (ASF) outbreak and intensive nationwide hunting for preventative ASF control since 2019. The hunting bag of wild boar in 2019 and 2020 was 99,920 and 97,043, respectively, which is almost twice as high compared to previous years. In addition, ASF outbreaks have been reported to reduce the infected population by approximately 90% in affected regions [28], potentially having a greater impact on the population than hunting. The consequences of hunting and the ASF outbreak have been reflected in the population density estimates of wild boar, which had been constantly increasing from 5/km^2^ in 2015 to 5.6/km^2^ in 2017 and to 6/km^2^ in 2019, having nearly halved in 2020 to 3.3/km^2^ and continuing to decrease according to latest reports (1.1/km^2^ in 2022) [29]. Hence, the abrupt lowering of *T. gondii* seroprevalence in 2020 may be explained by the recent perturbation of the wild boar population throughout the country, affecting the exposure and uptake of wild boars to *T. gondii* oocysts.

Another pattern observed in this study was the seasonal variation observed in wild boars, with the highest seroprevalence observed in fall and winter. Considering the seasonal breeding cycle of wild rodents and felines, previous studies proposed the highest number of susceptible felines being present during fall, which would lead to the highest exposure and shedding in these consecutive seasons [1,30,31]. With the accumulation of oocysts in the environment and their high survival, the exposure of intermediate hosts, such as ungulates, to these oocysts is also suspected to peak from the end of fall through winter [32]. Although this may explain the highest seroprevalence of *T. gondii* being observed in both species during winter in this study, the outcome suffers from unequal numbers of samples by season and the small overall number of samples when divided by season and year. Year and season should be simultaneously analyzed considering the impact of meteorological factors such as rainfall and temperature on the seasonality of *T. gondii* oocyst survival [19,32,33]. Therefore, further studies with similar sample sizes across seasons and years will be necessary to better clarify the association between seasonality and the infection of wild ungulates with *T. gondii*.

The seroprevalence of *T. gondii* in wild boar rose with increasing body mass, where a seroprevalence of 47.9% was observed in individuals weighing over approximately 300 kg. Given the strong association between body mass and age in wild boar, both factors are known to be good predictors regarding previous exposure to the parasite [25,34]. A higher seroprevalence of *T. gondii* antibodies in older animals has been reported in previous studies, which may be explained by increased encounters with the parasite, although the persistence of antibodies to *T. gondii* in wild boar is yet undetermined [19,35,36].

In the case of KWD, the seroprevalence of *T. gondii* (29.9%) was similar to previous reports conducted in wild cervids, most commonly in roe deer, red deer, and white-tailed deer, ranging from approximately 15 to 45% [37]. Among cervids, the prevalence observed in this study falls in the upper range of overall prevalence, close to observations in roe deer and white-tailed deer, which may be related to the synanthropic nature of these species [37,38]. KWD utilize habitats near agricultural landscapes, foraging on crops, especially in seasons where natural food sources are scarce [39,40], which may increase the habitat overlap with free-roaming cats living in the vicinity of rural residents, leading to higher exposure to *T. gondii* oocysts contaminating the environment.

The increase in T.* gondii* seroprevalence in KWD between 2019 and 2020 may be the result of heightened hunting activity throughout the country from 2019 onwards. Hunters and hunting dogs have been known to perturb the space use and/or behavior of non-target species [41,42]. Disturbed ranging behavior and movement outside of their stable home range potentially increased the chances for animals to be exposed to parasites transmitted through environmental contaminants, such as *T. gondii* [43].

In both species, sex did not have a significant association with *T. gondii* seroprevalence, although it was higher in males in both wild boar and KWD. This shared pattern is likely related to the difference in the size of the home range and/or the movement distance between the sexes, but an insignificant association between *T. gondii* seroprevalence and sex has been frequently reported in previous studies [7,25,44].

It should be noted that the results of serological analysis, such as in this study, provide information regarding the history of individuals in relation to *T. gondii* infection, rather than the existence of oocysts in the tissue at the time point of sampling.

## 5. Conclusions

In summary, the findings from this study highlight the widespread distribution of *T. gondii* infections in both of the wild ungulates in the ROK and reconfirms the risk posed to public health by consuming undercooked meat of hunted game species, especially wild boar [5]. The current nationwide population control measures and the monitoring of wild boars may provide valuable opportunities to identify the fluctuation in parasite infections with a density-dependent transmission mode, such as *T. gondii*, in wild boars. The continuous surveillance of *T. gondii* prevalence in wild boars and analyzing its association with various risk factors such as population density, rainfall/temperature, and the occurrence of feline hosts in the area will allow the clarification of drivers behind the patterns of *T. gondii* infection.

## Figures and Tables

**Table 1:** Seroprevalence for *Toxoplasma gondii* in wild boars and Korean water deer.

Species	Variable	Category	Sample Number	*T. gondii* Prevalence (%)	Odds Ratio (95% CI)	*p*-Value (*p* < 0.05)
Wild boar	Sex	Female	243	32.1	-	0.23
		Male	244	37.7	1.28 (0.88, 1.86)	
	Body mass	<20 kg	129	17.8	-	0.00
		20–60 kg	149	36.9	2.69 (1.54, 4.72)	
		60–100 kg	115	40.9	3.18 (1.78, 5.71)	
		100–300 kg	94	47.9	4.23 (2.31, 7.76)	
	Year	2017	175	41.7	-	0.00
		2019	157	41.4	0.99 (0.64, 1.53)	
		2020	155	20.6	0.36 (0.22, 0.59)	
	Season	Spring	61	39.3	0.84 (0.45, 0.58)	0.00
		Summer	211	24.6	0.42 (0.27, 0.66)	
		Fall	161	43.5	-	
		Winter	54	44.4	1.04 (0.56, 1.93)	
	Total		487	34.9		
Korean water deer	Sex ^1^	Female	114	29.8	-	0.43
		Male	130	35.4	1.29 (0.75, 2.21)	
	Year	2017	176	27.8	-	0.27
		2018	125	29.6	1.09 (0.66, 1.81)	
		2019	83	26.5	0.93 (0.52, 1.68)	
		2020	68	39.7	1.70 (0.95, 3.07)	
	Season	Spring	149	30.2	1.30 (0.67, 2.52)	0.82
		Summer	202	30.7	1.33 (0.70, 2.52)	
		Fall	64	25.0	-	
		Winter	37	32.4	1.44 (0.59, 3.51)	
	Total		452	29.9		

^1^ Samples lacking sex information were excluded from the analysis.

**Table 2:** Samples analyzed in this study per province. The names of provinces are as follows; Gyeonggi (GG); Gangwon (GW); Chungnam (CN); Chungbuk (CB); Jeonbuk (JB); Jeonnam (JN); Gyeongbuk (GB), Gyeongnam (GN), Jeju (JJ).

Province	Wild Boar		KWD	
	Sample Number	*T. gondii* Prevalence (%)	Sample Number	*T. gondii* Prevalence (%)
GG	133	30.8	44	18.2
GW	164	31.1	88	34.1
CB	26	42.3	12	0.0
CN	16	25.0	77	29.9
JB	25	28.0	56	30.4
JN	21	61.9	42	33.3
GB	56	42.9	37	21.6
GN	43	41.9	95	35.8
JJ	3	33.3	1	100.0

## Data Availability

The raw data that were analyzed in this article are available upon direct request from the authors.
